# Limitations of Stroke Volume Estimation by Non-Invasive Blood Pressure Monitoring in Hypergravity

**DOI:** 10.1371/journal.pone.0121936

**Published:** 2015-03-23

**Authors:** Olivier Manen, Caroline Dussault, Fabien Sauvet, Stéphanie Montmerle-Borgdorff

**Affiliations:** 1 Percy Military Hospital, Aeromedical Centre, Clamart, France; 2 Armed Forces Biomedical Research Institute (IRBA), Brétigny-sur-Orge, France; 3 EA7330 VIFASOM, Paris Descartes University, Hotel Dieu, Paris, France; National University of Singapore, SINGAPORE

## Abstract

**Background:**

Altitude and gravity changes during aeromedical evacuations induce exacerbated cardiovascular responses in unstable patients. Non-invasive cardiac output monitoring is difficult to perform in this environment with limited access to the patient. We evaluated the feasibility and accuracy of stroke volume estimation by finger photoplethysmography (SVp) in hypergravity.

**Methods:**

Finger arterial blood pressure (ABP) waveforms were recorded continuously in ten healthy subjects before, during and after exposure to +Gz accelerations in a human centrifuge. The protocol consisted of a 2-min and 8-min exposure up to +4 Gz. SVp was computed from ABP using Liljestrand, systolic area, and Windkessel algorithms, and compared with reference values measured by echocardiography (SVe) before and after the centrifuge runs.

**Results:**

The ABP signal could be used in 83.3% of cases. After calibration with echocardiography, SVp changes did not differ from SVe and values were linearly correlated (p<0.001). The three algorithms gave comparable SVp. Reproducibility between SVp and SVe was the best with the systolic area algorithm (limits of agreement −20.5 and +38.3 ml).

**Conclusions:**

Non-invasive ABP photoplethysmographic monitoring is an interesting technique to estimate relative stroke volume changes in moderate and sustained hypergravity. This method may aid physicians for aeronautic patient monitoring.

## Introduction

Aeromedical evacuations are consistently increasing in both military and civilian contexts [1,2]. Many war casualties are evacuated to a military hospital in Europe, shortly after resuscitation and damage control surgery on the theatre of operations. Long aeromedical evacuations of unstable patients are common [1,3].

Patients lying onboard transport aircrafts or onboard airliners encounter inertial forces in the head-to-foot direction, i.e. Gz accelerations, mostly during the take-off and landing [4]. The Federal Aviation Authority defines a maximum range of −1.0 to +2.5 Gz for transport category airplanes [5]. In case of turbulence, accelerations can reach 3 G in the x, y and z axes. +Gz accelerations are responsible for leg blood pooling, which decreases venous return to the heart, preload and following stroke volume (SV), cardiac output (CO) and arterial blood pressure (ABP) [6,7]. These effects are compensated by baroreflex activation after ∼10s in healthy humans. Such a reaction cannot be expected in a patient with a severe blood loss [3]. In a recent study on aeromedical evacuations of patients with severe battle injuries, Dubost et al. observed changes in cardiac output from the take-off to the 50^th^ min of flight [3].

Consequently, SV and ABP monitoring of unstable evacuated patients is vital. It should preferably be performed non-invasively for a better patient tolerance and less risks of damage when turbulence is present. Validated standards for CO and SV measurement are thermodilution, rebreathing [8] and echocardiography [9–11]. However, these techniques have been validated in stable conditions and allow only discontinuous measurements. They would not detect rapid changes. As an alternative, algorithms have been developed to estimate SV and CO from ABP [10–12]. The most reliable algorithms are Liljestrand, the systolic area method and Windkessel [9,13].

The changes in relative SV or CO estimated by finger photoplethysmography have been evaluated in healthy subjects and Intensive Care Unit patients, and compared to the validated standards [9,14–20]. However, the validity of these studies is questioned [10,21]. In particular, SV and CO determination by photoplethysmography has not been compared to echocardiographic measurements in unstable environments. In addition, the possible deleterious effect of accelerations on ABP signal monitoring by photoplethysmography has never been studied.

The aim of this study was to evaluate the feasibility and accuracy of SV estimation by finger photoplethysmography in comparison to echocardiography, in a situation of hypergravity constraint: before and after exposure to +Gz accelerations. The second objective was to determine which algorithm was the most accurate for SV computation in this situation: Liljestrand, Windkessel or the systolic area method.

## Materials and Methods

### Subjects

Ten healthy male military volunteers participated in the study (age 30 ± 2 years, body surface 1.95 ± 0.20 m^2^). They were non-smokers and free from heart, vascular or lung disease, ECG or echocardiographic abnormalities and took no medication. The Ethics Committee Ile-de-France III and the French National Agency for Drug Safety approved of the protocol (approvals number 2739 and 2009-A01092-55 respectively). All subjects gave their written informed consent.

### Human centrifuge

Experiments were carried out in the human centrifuge of the Armed Forces Biomedical Research Institute (IRBA) in Brétigny-sur-Orge, France (model 101.3; Latecoère, France). In this study, the backrest angle of the gondola seat was 32° (pilot seat MK10; Martin Baker, Great Britain). Four EL 503-10 ECG electrodes (Biopac Systems; Goleta, CA, USA) were placed on the subject’s chest for monitoring of ECG (DI and DII leads).

### Equipment

Subjects wore a flying suit and size-fitted anti-G trousers (ARZ 825; Aérazur, France) to prevent G-intolerance during the long centrifuge runs. Trousers inflated automatically from 10 hPa at +2 Gz with a gain of 70 hPa/Gz.

Plethysmographic arterial beat-by-beat blood pressure was recorded from the left third finger by a volume-clamp technique (PortaPres FMS; Finapres Medical Systems BV, The Netherlands) [16,22]. This device was calibrated against a reference value on the right arm measured with a manual sphygmomanometer, at the onset and end of each echocardiography. The height difference between the digital sensor and the heart was corrected by putting the reference electrode at cardiac level. Echographic measurements were performed using a portable echocardiograph (MyLab30CV; Esaote, France) and a 2.5 MHz probe.

### Protocol

**Fig 1:**
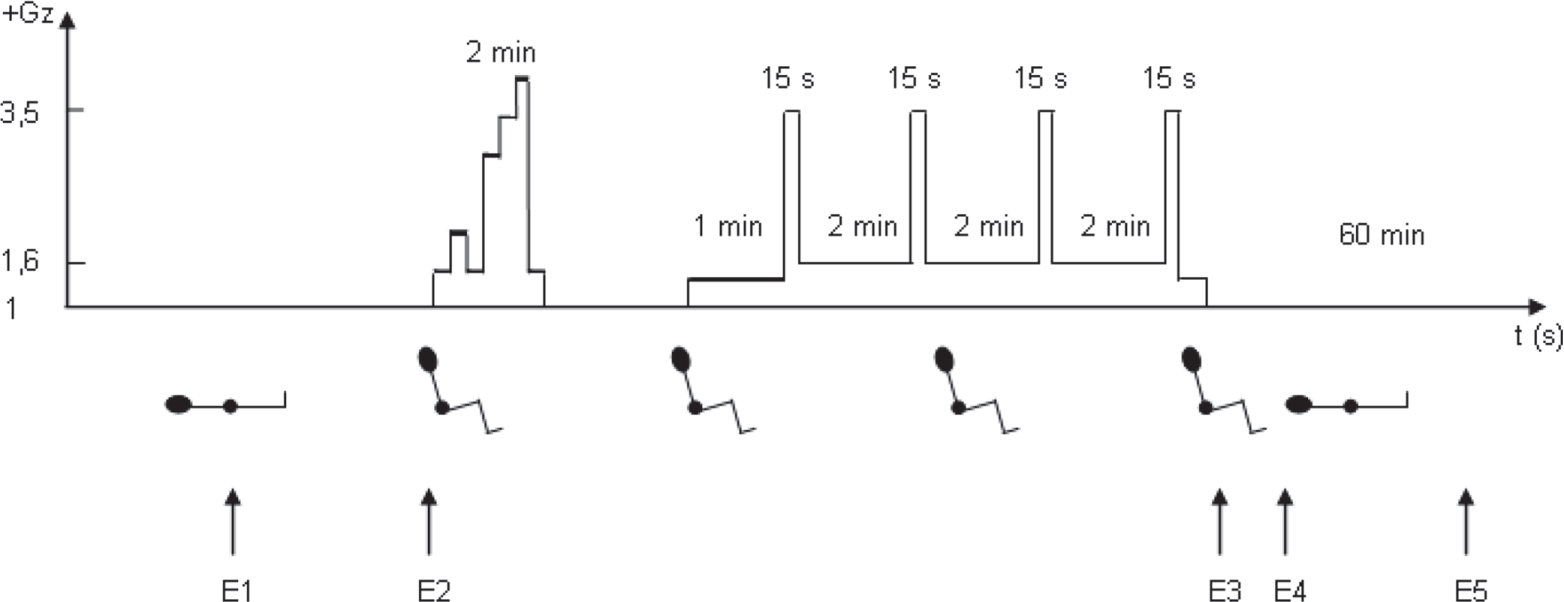
Schematic representation of the protocol. The subject, equipped with anti-G trousers, was exposed to +Gz accelerations during two centrifuge runs (total exposure time: 10 min). Arterial blood pressure was recorded by finger photoplethysmography continuously. Echocardiographic measurements were performed before the runs (E1, supine left lateral, E2, sitting with a 32° backrest angle in the centrifuge gondola), immediately after (E3, sitting in the gondola, E4, supine left lateral) and after 60 min of recovery (E5, supine left lateral). N = 10.

The study protocol is shown in Fig. 1. The ten subjects were naïve to the centrifuge, they didn’t have any experience for piloting an aeroplane, to be compared to real patients. The first echocardiography (E1) was performed in the left lateral supine posture after a 10-min supine rest.

The subject was then equipped and seated in the centrifuge gondola. A second echocardiography (E2) was performed after 10 min of sitting rest. The centrifuge was then started and two runs were performed: 1) a two-min warming-up run, which reached +4 Gz, and 2) an eight-min sustained-Gz run composed of three two-min periods at +1.6 Gz and four 15-s plateaus at +3.5 Gz. The jolt was +0.5 Gz.s^−1^ during the warming-up and +1 G.s^−1^ during the second run. The range and variations of +Gz accelerations during these runs were chosen to be in accordance with those observed onboard transport aircrafts, and also to optimize the stable data acquisition. During Gz exposure, the subjects were told not to perform muscle-tensing—i.e. static contractions of the arm, abdominal and leg muscles—combined with a Valsalva manoeuvre (M1 anti-G straining manoeuvre used to sustain high-Gz levels). This would have modified the finger blood flow.

The third echocardiography (E3) was performed immediately after the completion of the centrifuge runs, with the subject still sitting in the gondola seat. He was then transported supine on a gurney and the fourth echocardiography (E4) was performed in the left lateral supine posture about 10 min later. After 60 min of supine rest, the last echocardiography (E5) occurred in the same posture.

Great care was taken to ensure the quality of ABP monitoring and acquisition: the ambient temperature was constantly above 22°C except for time point E3 (20.2°C), and the subject’s hand carrying the finger cuff was covered with a blanket as recommended by Imholz et al [23]. The local circulation was stimulated when necessary [24]: several subjects had cold fingers, which had to be regularly dipped in warm water during the protocol; during +Gz-exposure, the subjects were asked to move their fingers regularly to improve the signal quality.

Each echocardiography lasted five to ten minutes.

### Collection and analysis of data

ABP waveform was recorded with a data-handling system (Biopac Systems; Goleta, CA, USA) at 625 Hz sampling frequency and stored digitally. Analysis was performed at the time points of the echocardiographies with a dedicated program created with the MatLab software (MatLab 7.3.0.267-R2006b; The Mathworks, USA). 300-s ABP waveform segments were selected, according to the procedure described by Lu et al [1]. Algorithms for the calculation of beat-by-beat SV were applied as follows: pulse pressure (Windkessel), systolic area (Systolic Area) and pulse pressure / (systolic arterial pressure + diastolic arterial pressure) (Liljestrand & Zander) [11,13]. CO was calculated as CO = Heart Rate x SV. One mean SV (relative value) was computed per ABP segment and subject: SVp(relative).

The echocardiographic data stored when possible were the following: parasternal view, sub-aortic diameter; apical view, sub-aortic velocity-time integral (VTI); supra-sternal view, aortic VTI (MyLab Desk 4.03 software, Esaote, France). The analysis was performed by two independent operators. For each echocardiography, the three best sub-aortic diameter recordings were selected and used to compute one mean sub-aortic diameter and surface (S). The three best VTIs were also selected. Corresponding SV were calculated as SV = S x VTI. One mean SV (absolute value) was computed per echocardiography and subject: SVe.

In order to enable comparison between SVe and SVp, a calibration factor K was defined at E1 as K = SVe / SVp(relative) [11]. Absolute values for SVp were computed at E2, E3, E4 and E5 as SVp(absolute) = K x SVp(relative). For convenience, in the remainder of this manuscript SVp will refer to SVp(absolute).

In addition, mean values of systolic and diastolic ABP, Heart Rate, SVp and CO were computed in hypergravity (one mean value per plateau) for all subjects together.

### Statistics

The analyses were made using STATISTICA 8 (Statsoft; Maisons Alfort, France).

For all parameters, a repeated-measure ANOVA with two factors, time (E1 to E5) and method (SVe, SVp(Liljestrand), SVp(Systolic Area) and SVp(Windkessel)) was performed. Significance was accepted at p<0.05. When the results were significantly different, a post-hoc Bonferroni test was applied for all pairwise comparisons.

A Bland–Altman plot was employed to assess repeatability between the two methods. The limit of agreement (LOA) was calculated as the mean of differences ±1.96 SD (Bland and Altman, 1986) [25]. Data are presented as means ± SD.

Intra-class correlations between SVe and SVp and 95% confidence intervals were calculated with the three algorithms [26].

Correlation strength within each method was determined by correlation coefficient calculation and regression analysis. The slope of the relationship between the methods as well as the intercept were compared to the reference equation (y = x) using the ANCOVA analysis.

No statistics were performed on data recorded in hypergravity, as SVp could not be compared to SVe in these conditions and time courses of ABP, SV and CO were in accordance with the existing literature on the topic [6,27,28].

## Results

The tolerance of the subjects during the centrifuge runs was excellent, with no greyout, blackout or discomfort related to the anti-G trousers.

### Quality of the photoplethysmography signal

16% of the ABP segments concomitant with the 50 ultrasound measurements were not exploitable, particularly during E5 (50%). The main issue was discrepancies between Portapres and sphygmanometer BP values (63%); other problems were BP fluctuations (25%) and a technical default (12%). Inappropriate Portapres values were either too high (three segments, mean difference ± SD, 35.2 ± 3.8 / 9.8 ± 8.9 mmHg) or too low (two segments, 12.3 ± 4.6 / 21 ± 2.1 mmHg). Segments presenting an acceptable signal-noise ratio were selected.

300-s intervals could be retrieved in 41 out of 42 exploitable segments, with a 230-s interval in the 42nd.

### Effect of +Gz accelerations

**Fig 2:**
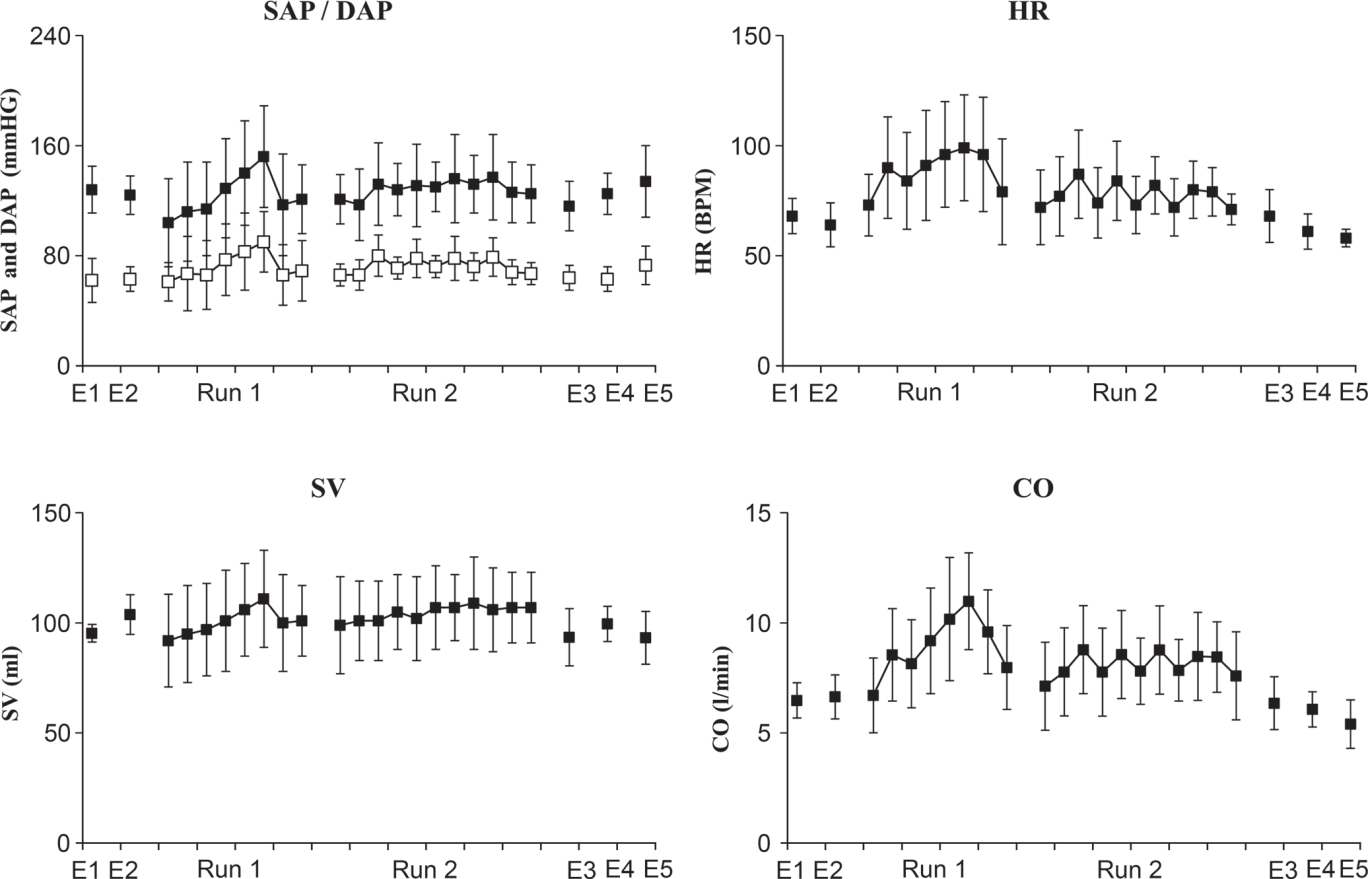
Time course of cardiovascular parameters measured by or computed from finger arterial blood pressure (ABP). Parameters measured: SAP, systolic arterial pressure; DAP, diastolic arterial pressure; Parameters computed: HR, heart rate; SV, stroke volume; CO, cardiac output.

During the warm-up run, a decrease in SV was observed at the onset of hypergravity exposure, with an increase in Heart Rate and no change for CO. Then, exposure to +Gz accelerations led to a gradual increase in systolic and diastolic ABP, Heart Rate, SV and CO in all subjects. During the second run, inter-individual variability was higher, even though the general tendency was comparable. Mean values are shown in Fig. 2.

SV was computed using the Liljestrand algorithm. CO is defined as SV x HR. Mean values were assessed during E1, E2, E3, E4 and E5, during the first and the second runs of +Gz-accelerations.

### Reproducibility

**Fig 3:**
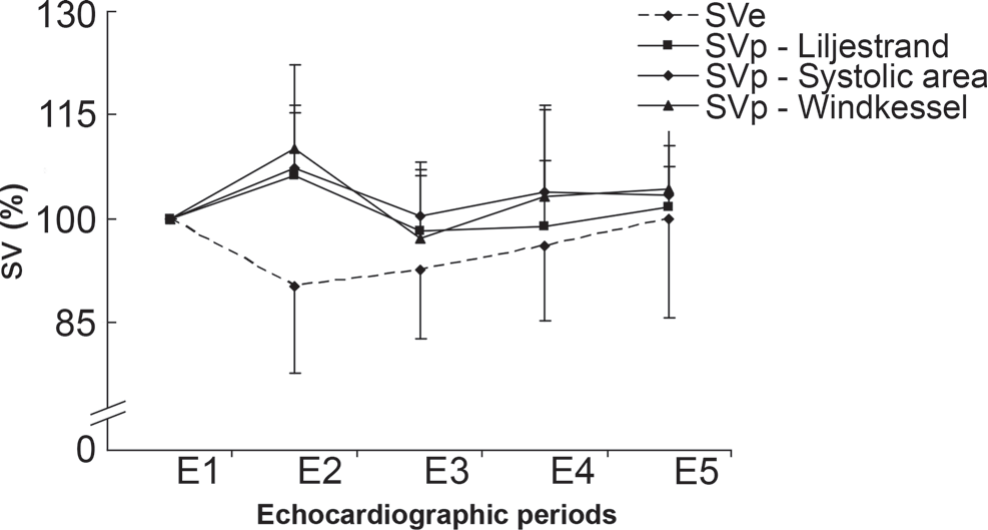
Stroke volume (SV) computed from echocardiographic and finger arterial blood pressure recordings (SVe and SVp respectively), at the time points described in Fig. 1. Three algorithms were used for SVp computation: Liljestrand, the systolic area and Windkessel.

SVp and SVe are presented in Table 1. SVe results were comparable between the two operators. SVp did not differ from SVe (p = 0.07). There was no difference between SVp calculated with the three algorithms either (p = 0.23) (Fig. 3). No effect of time on SV was observed (p = 0.4). Contrastingly, there was an interaction between method and time (p = 0.01). This discrepancy was due to a difference between SVp and SVe at time point E2 in the Bonferroni post-hoc test (p = 0.024).

**Table 1:** Mean differences and limits of agreement for the Bland-Altman plots.

	SVp computation algorithms			SVe
	Liljestrand	Windkessel	Systolic area	
**SV_mL_**	92.4 ± 16.2	94.4 ± 16.3	94.9 ± 17.1	86 ± 19.8
**(SVp-SVe)_mL_**	6.5	8.5	8.9	
**(SVp-SVe)_%_**	7	9	10	
**Limits of agreement between SVe and SVp (Bland-Altman)_mL_**	−22.8; 35.7	−22.9; 39.9	−20.5; 38.3	
**Limits of agreement between SVe and SVp (Bland-Altman)_%_**	−26; + 40	−25; + 44	−23; + 42	

**Fig 4:**
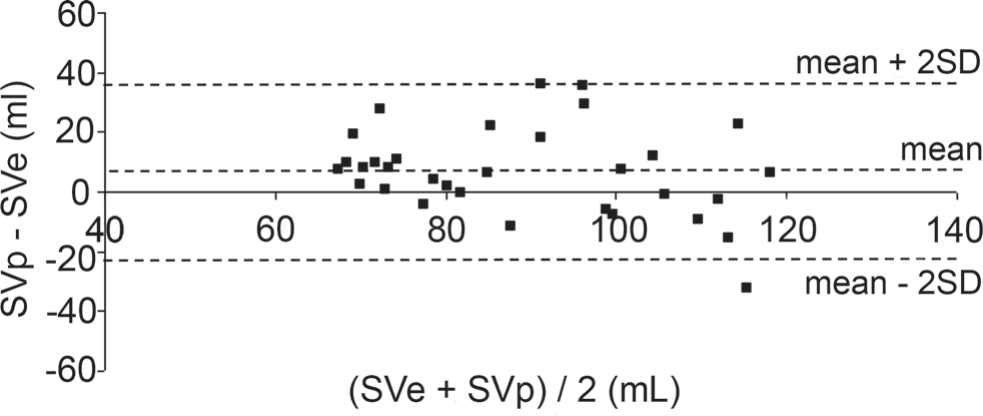
Bland-Altman plots for determination of agreement between SVe and SVp computed with the Liljestrand algorithm. ----: limits of agreement (maximum, minimum, mean).

**Fig 5:**
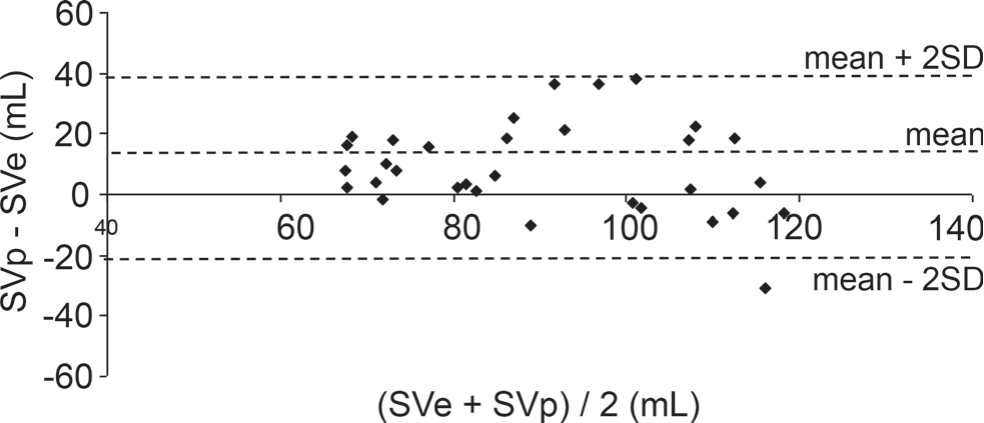
Bland-Altman plots for determination of agreement between SVe and SVp computed with the systolic area method. ----: limits of agreement (maximum, minimum, mean).

**Fig 6:**
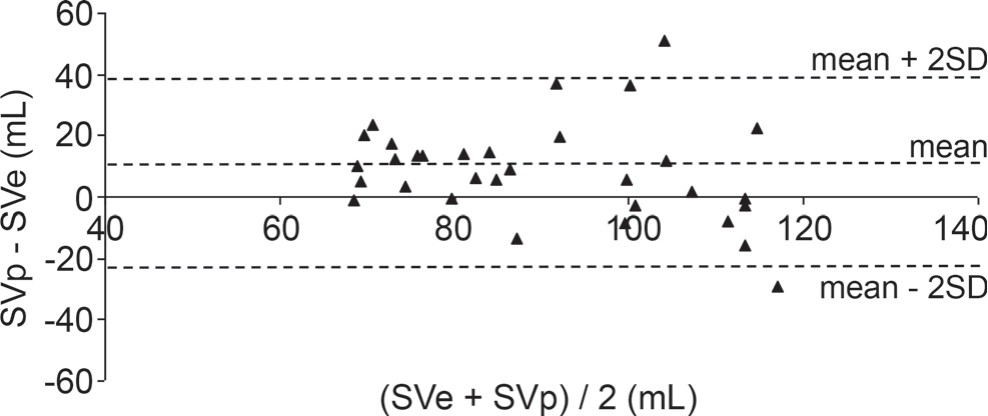
Bland-Altman plots for determination of agreement between SVe and SVp computed with the Windkessel algorithm. ----: limits of agreement (maximum, minimum, mean).

The differences between SVp and SVe plotted against their mean value according to Bland and Altman are shown in Figs. 4–6. Mean differences and limits of agreement (LOA) are presented in Table 1. %LOAs for the Liljestrand, Windkessel and systolic area algorithms were 93.7%, 93.7% and 96.9%, respectively.

### Comparison between methods

Intra-class correlations between SVe and SVp and 95% confidence intervals (ICC (CI 95%)) were 0.71 (0.54–0.81) for Liljestrand, 0.68 (0.47–0.80) for Windkessel and 0.70 (0.50–0.81) for the systolic area method.

**Fig 7:**
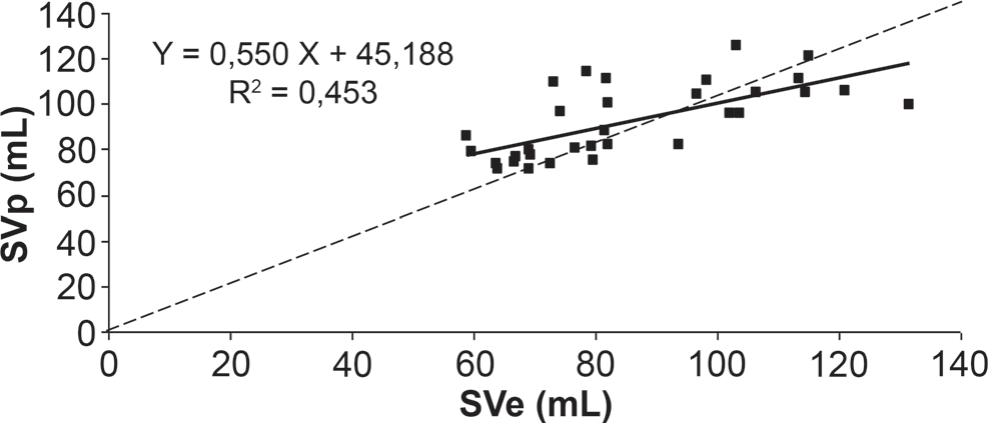
Correlation plots between SVe and SVp computed with the Liljestrand algorithm. ----: equality line.

**Fig 8:**
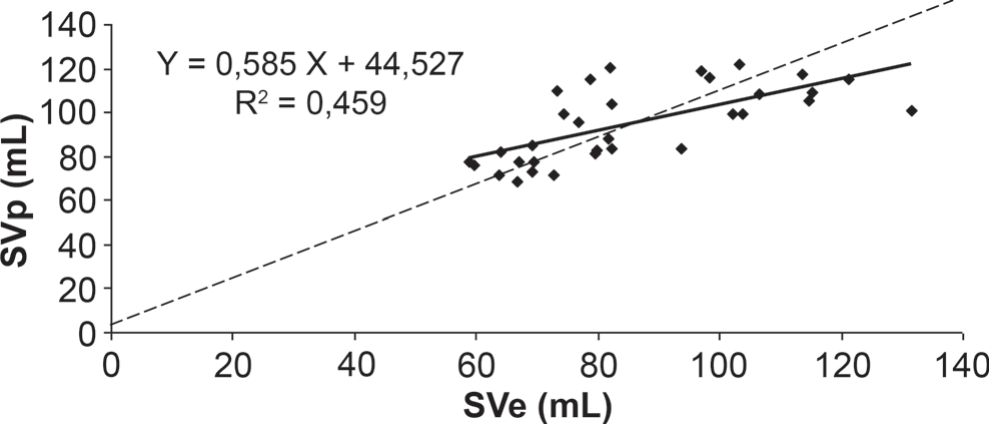
Correlation plots between SVe and SVp computed with the systolic area method. ----: equality line.

**Fig 9:**
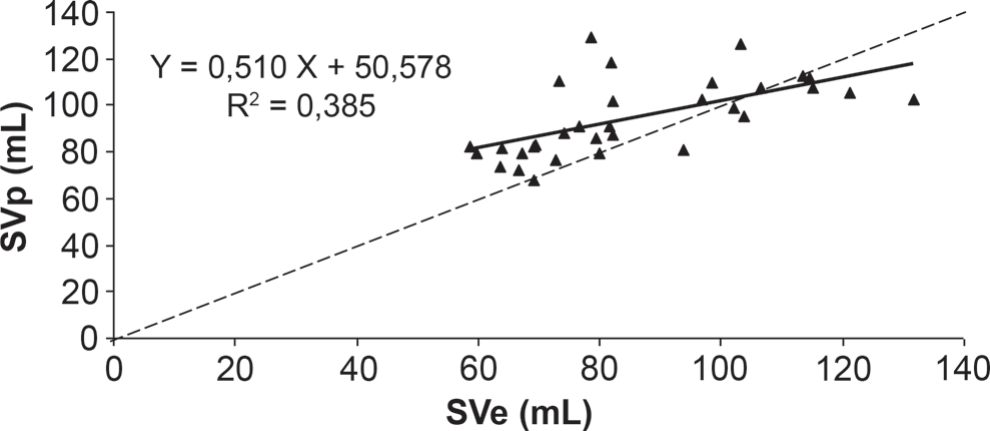
Correlation plots between SVe and SVp computed with the Windkessel algorithm. ----: equality line.

The correlation between SVp and SVe (Figs. 7–9) was significant for the three algorithms (p<0.001). The correlation coefficient was 0.67 for Liljestrand, 0.62 for Windkessel and 0.68 for the systolic area method. Regression slopes did not differ from the equality line for all three algorithms (Liljestrand, F(1,60) = 1.36, p = 0.24; Windkessel, F(1,60) = 2.38, p = 0.13; systolic area, F(1,60) = 2.22, p = 0.14). However, Y-axis intercepts were different for the three models (Liljestrand, F(1,60) = 5.06, p = 0.03; Windkessel, F(1,60) = 7.09, p = 0.01; Systolic area, F(1,60) = 8.97, p = 0.004).

## Discussion

Photoplethysmography is one of the few non-invasive operator independent techniques that may enable CO monitoring in unstable environments. For the first time, this study demonstrates that SV and CO can be estimated by photoplethysmography in hypergravity in healthy subjects, during sustained exposures up to +4 Gz. There is a good agreement between SV changes estimated by photoplethysmography and by echocardiography used as the reference technique.

However, before the centrifuge runs (time point E2) we observed a discrepancy between photoplethysmographic and echographic SV. This implies that the calibration with echocardiography must be performed regularly if this device is available. Photoplethysmography used alone is validated to follow SV trends only.

An additional result is that the three SVp computation algorithms gave comparable values. Reproducibility was the best for the systolic area algorithm.

### Feasibility of SV determination during +Gz accelerations

#### ABP measurement

The present study shows that a continuous ABP measurement is feasible in hypergravity and in a restrained environment, if the signal is frequently checked and calibrated. ABP signal quality was mostly good (84% of recordings) albeit 16% of photoplethysmographic recordings could not be analyzed. +Gz accelerations were not responsible for bad signal quality, as most discarded recordings belonged to the last supine rest period. The software detected signal variations and loss but not drifts from the reference sphygmomanometer measurements.

Photoplethysmography has been used occasionally in short-term and low level hypergravity (20–25 s) for ABP measurement [29,30] and SV determination [31]. Signal quality is not discussed in these studies. We found no reports of photoplethysmography being used during sustained hypergravity higher than +2 Gz.

Photoplethysmographic ABP has been compared to invasive (intra-arterial) and non-invasive (sphygmomanometry) reference methods. A good correlation was found at rest in the sitting [32–34] and supine postures [32], but not when photoplethysmography was used for patient triage and monitoring in emergency ward [35]. During exercise, it overestimates systolic ABP and underestimates diastolic ABP [34,36]. In conclusion, photoplethysmographic ABP must be calibrated with a reference method in every situation that differs from a sustained rest.

#### Photoplethysmographic SV vs. echocardiographic SV

Echographic measurements were difficult to perform in the centrifuge gondola. The subject had to be moved for correct imaging, and the hand equipped with the finger cuff went frequently above the level of the heart. We skipped the supra-sternal view whose access was too uncertain.

ABP was corrected with sphygmomanometry before each echocardiography. Baseline SVp was then calibrated with baseline SVe. Despite these precautions, a constant overestimation of SVp compared to SVe was found in all postures. No information is available in hypergravity, as echocardiography could not be performed during the centrifuge runs. We observed that time courses of SVp and SVe were comparable during the protocol and that no difference was found between SVp computed with the three algorithms. These findings imply that ABP waveforms can only be used for SV determination if the measurement accuracy is frequently questioned by comparing with the subject or patient’s clinical state and sphygmomanometer measurements.

The agreement was good between SVp and SVe except for time point E2. We have no explanation for this discrepancy as far as suggestions can been proposed for SVp and SVe variations between E1 and E2: on the one hand, SV was expected to fall down when the subject went from supine to sitting posture, due to a decreased cardiac venous return, and so SVe at E2 could be more accurate than SVp; on the other hand, we may consider the stress of the subject sitting in the gondola before the centrifuge runs, which could increase the breathing frequency, the finger blood flow and consequently SVp.

### Effect of +Gz accelerations on ABP, Heart Rate, SV and CO

In the present study, the subjects were fit and healthy. They tolerated the long runs well, even though they complained of fatigue at the end of the protocol. ABP recordings show that the baroreflex reacted almost immediately to hypergravity. For these subjects naïve to the centrifuge, the first contact with +Gz-accelerations resulted in an exacerbated baroreflex reaction, which tended to be more adapted during the second run. The recovery occurred quickly after the centrifuge stop. Indeed, regular exposure to hypergravity increases G-tolerance [37–39]. Whether unstable patients like those studied by Dubost et al. [3] would adapt so well to such conditions is doubtful.

Hypergravity up to +3 Gz is usually well tolerated [40], so we did not expect the large decreases in ABP, SV and CO described by Lindberg et al [6]. In addition, the moderate jolt gave the baroreflex time to react and the backrest seat angle lowered +Gz by cos32°. Furthermore, anti-G trouser inflation increased preload, SV and afterload [7,29,41].

### Limitations of the study

We chose to use echocardiography as the reference technique. However, the inter-operator variability of end-systolic volume determination is 13.7–20.3% for echocardiography, compared to 4.4–9.2% for magnetic resonance imaging, the golden standard in non-invasive cardiac imaging [24,42]. This high variability of the echocardiographic method could explain the large LOAs [25,43] (i.e. -21;+38 mL) obtained with the three algorithms. However, echocardiography is the only non-invasive technique which enables SV determination in a confined environment such as the centrifuge gondola.

The main limitation of the protocol was the impossibility to use echocardiography during the centrifuge runs. Actually, the only possible way of determining CO in hyper- or microgravity environment is by rebreathing [28,29,44]. Rebreathing is a golden standard for CO determination. Yet, we chose not to use it as the reference because it is unsuitable for routine use during aeromedical evacuations: rebreathing requires a voluntary breathing manoeuvre from the subject. Contrastingly, portable echocardiographs do not require the patients’ participation.

### Conclusions

Practitioners in charge of aeromedical evacuations are interested in compact, easy-to-use and readily available techniques of measurement. Finger photoplethysmography corresponds with these criteria. The present study shows that estimating SV changes from ABP waveforms is acceptable in extreme conditions such as exposure to hypergravity in a human centrifuge. The systolic area method is the most reproducible of the three algorithms tested in these conditions. These results can be used as a basis for comparison with other new techniques in future studies on non-invasive SV monitoring in the aeronautical environment.

## Supporting Information

S1 Dataset SVe computation. Ultrasound data: SAD, sub-aortic diameter; VTI, sub-aortic velocity-time integral. Parameter computed: SVe. Periods: DLG1, E1; NAC1, E2; NAC2, E3; DLG2, E4; REC, E5.
(XLS)

S2 Dataset SVp(absolute) computation with the three algorithms. A factor K was defined at E1 as K = SVe / SVp(relative); SVp(absolute) was computed at E2, E3, E4 and E5 as SVp(absolute) = K x SVp(relative). SVp R, SVp(relative); SVp A, SVp(absolute).
(XLS)
